# A Phylogenetic and Taxonomic Study on *Xylodon* (Hymenochaetales): Focusing on Three New *Xylodon* Species from Southern China

**DOI:** 10.3390/jof8010035

**Published:** 2021-12-30

**Authors:** Meng-Han Qu, Dong-Qiong Wang, Chang-Lin Zhao

**Affiliations:** 1 Key Laboratory for Forest Resources Conservation and Utilization in the Southwest Mountains of China, Ministry of Education, Southwest Forestry University, Kunming 650224, China; fungimenghanq@163.com (M.-H.Q.); fungidongqiong@163.com (D.-Q.W.); 2 College of Forestry, Southwest Forestry University, Kunming 650224, China; 3 College of Biodiversity Conservation, Southwest Forestry University, Kunming 650224, China; 4 Yunnan Key Laboratory for Fungal Diversity and Green Development, Kunming 650201, China; 5 School of Life Sciences, Tsinghua University, Beijing 100084, China

**Keywords:** biodiversity, *Hyphodontia* s.l., molecular systematics, white-rot fungi, macro-fungi, Yunnan-Guizhou Plateau

## Abstract

Three wood-inhabiting fungal species, *Xylodon laceratus*, *X. montanus,* and *X. tropicus* spp. nov., were collected from southern China, here proposed as new taxa based on a combination of morphological features and molecular evidence. *Xylodon laceratus* is characterized by the resupinate basidiomata with grandinioid hymenophore having cracked hymenial surface, and ellipsoid basidiospores; *X. montanus* is characterized by the annual basidiomata having the hard, brittle hymenophore with cream hymenial surface, and ellipsoid to broadly ellipsoid basidiospores (3.9–5.3 × 3.2–4.3 µm); and *X. tropicus* is characterized by its grandinioid hymenophore with buff to a pale brown hymenial surface and subglobose basidiospores measuring 2–4.8 × 1.6–4 µm. Sequences of ITS and nLSU rRNA markers of the studied samples were generated, and phylogenetic analyses were performed with maximum likelihood, maximum parsimony, and Bayesian inference methods. The ITS+nLSU analysis of the order Hymenochaetales indicated that the three new species clustered into the family Schizoporaceae, located in genus *Xylodon*; based on further analysis of ITS dataset, *X. laceratus* was a sister to *X. heterocystidiatus*; *X. montanus* closely grouped with *X. subclavatus* and *X. xinpingensis* with high support; while *X.* *tropicus* was retrieved as a sister to *X. hastifer*.

## 1. Introduction

Wood-inhabiting fungi are noteworthy components of woody plant ecosystems and take an active part in the decomposition and turnover of nutrients from wood, in which the corticioid fungi are one of the major groups of wood-inhabiting Basidiomycota [1]. Hymenochaetales is one of the most important orders in Basidiomycota because many species in this order are medicinal fungi, and some of them are forest pathogens [2]. Within Hymenochaetales, commonly accepted families Chaetoporellaceae, Coltriciaceae, Hymenochaetaceae, Hyphodontiaceae, Neoantrodiellaceae, Nigrofomitaceae, Oxyporaceae, and Schizoporaceae are supported as individual monophyletic lineages [3]. Schizoporaceae was introduced by Walter Jülich with *Schizopora* Velen. as the type genus and included the other three genera *Fibriciellum* J. Erikss. & Ryvarden, *Fibricium* J. Erikss. and *Fibrodontia* Parmasto [4]. 

The genus *Xylodon* (Pers.) Gray (Schizoporaceae, Hymenochaetales), with the generic type *X. quercinus* (Pers.) Gray is characterized by the basidiomata having a smooth, tuberculate, grandinioid, odontioid, coralloid, irpicoid or poroid hymenophore, and a monomitic, pseudodimitic or dimitic hyphal system with clamped generative hyphae and mostly presence of 1 to 3 types cystidia, negative in Melzer’s reagent; barreled clavate or suburniform basidia, and globose to ellipsoid to cylindrical basidiospores, additionally causing a white rot [5,6]. It is the largest segregate genus of *Hyphodontia* J. Erikss. s.l. (Hymenochaetales, Basidiomycota) based on the MycoBank database (http://www.MycoBank.org, accessed on 30 November 2021) and the Index Fungorum (http://www.indexfungorum.org, accessed on 30 November 2021), both have registered 206 specific and infraspecific names in *Xylodon*, but the actual number of species reaches eighty-six [7,8,9,10,11,12,13,14,15,16,17,18,19,20,21,22,23,24,25,26,27,28,29,30,31,32].

The researches of *Xylodon* based on molecular systematics are coming to light from genomic analyses and environmental DNA surveys [33]. Species diversity of *Hyphodontia* sensu lato has been systematically surveyed in Europe for the last several decades, in which almost all taxonomic studies did not try to address the taxonomic status of species of *Hyphodontia* sensu lato at the family level, including the most important monograph “Die Gattung *Hyphodontia* John Eriksson” [14]. *Hyphodontia* s.l. could be split into 13 genera, of which the most species-rich ones are *Xylodon* and *Kneiffiella* P. Karst. However, due to a lack of rDNA sequences for many taxa, the molecular grounds were not enough to define separate genera clearly. Therefore the mycologists adopted a broad concept of *Hyphodontia* s.l. [25]. Molecular phylogeny analyses were performed by maximum parsimony and Bayesian methods, in which the phylograms from both analyses confirmed that the taxa from genera of *Xylodon*, *Schizopora*, *Palifer* Stalpers & P.K. Buchanan, *Lyomyces* P. Karst. and *Rogersella* Liberta & A.J. Navas were mixed within *Xylodon-Lyomyces-Rogersella* clade and *Xylodon-Schizopora-Palifer* clade [34]. Based on the morphological and/or phylogenetic information on *Hyphodontia* s.l., it was hard to differentiate between the genera *Xylodon* and *Schizopora*, with neither morphological nor molecular data and proposed that both genera should be united in one genus [22]. Used sequences from the ITS and nuclear LSU regions to infer the phylogenetic position of the *Xylodon*, confirmed *Lagarobasidium* Jülich as a synonym of *Xylodon*, in which three species *X. magnificus* (Gresl. & Rajchenb.) K.H. Larss., *X. pumilius* (Gresl. & Rajchenb.) K.H. Larss., and *X. rickii* (Gresl. & Rajchenb.) K.H. Larss. were transferred into *Xylodon* from *Lagarobasidium* [27]. Phylogenetic analyses of ITS and 28S sequences revealed that *Palifer* and *Odontiopsis* Hjortstam & Ryvarden should be synonymized under *Xylodon* and proposed four species new to science as *X. exilis* Yurchenko, Riebesehl & Langer, *X. filicinus* Yurchenko & Riebesehl, *X. follis* Riebesehl, Yurchenko & Langer, and *X. pseudolanatus* Nakasone, Yurchenko & Riebesehl [31]. The comprehensive phylogenetic analyses on the basis of multiple loci on *Hyphodontia* s.l. showed that the six genera were separated into four clades within Hymenochaetales, in which *Fasciodontia* Yurchenko & Riebesehl, *Lyomyces,* and *Xylodon* were accepted as members of a previously known family Schizoporaceae [3]. Maximum parsimony strict consensus tree illustrating the phylogeny of *Xylodon* species based on ITS sequences showed that *X. gossypinus* C.L. Zhao & K.Y. Luo grouped with *X. brevisetus* (P. Karst.) Hjortstam & Ryvarden, and *X. macrosporus* C.L. Zhao & K.Y. Luo was sister to *X. follis* and *X. sinensis* C.L. Zhao & K.Y. Luo formed two sister groups to *X. attenuatus* Spirin & Viner and *X. yarraensis* Xue W. Wang & L.W. Zhou with very low supports [32]. 

During investigations on wood-decaying fungi in southern China, three additional *Xylodon* taxa were found, which could not be assigned to any described species. The present study examines the taxonomy and phylogeny of the three new species within *Xylodon*. The current study focuses on the phylogenetic and taxonomic study of *Xylodon*, based on the internal transcribed spacer (ITS) and the large subunit nuclear ribosomal RNA gene (nLSU) sequences.

## 2. Materials and Methods

### 2.1. Sample Collection

Fresh fruiting bodies of the fungi were collected from the Dali, Puer, Wenshan, Yuxi of Yunnan Province, P.R. China. The dried specimens were dried in an electric food dehydrator at 40 °C, then sealed and stored in an envelope bag and deposited in the herbarium of the Southwest Forestry University (SWFC), Kunming, Yunnan Province, P.R. China.

### 2.2. Morphology

Macromorphological descriptions are based on field notes and photos captured in the field and lab. Color terminology follows Petersen [35]. Micromorphological data were obtained from the dried specimens after the observation under a light microscope with a magnification 10 × 100 oil [17]. The following abbreviations were used: KOH = 5% potassium hydroxide water solution, CB– = acyanophilous, IKI– = both inamyloid and indextrinoid, L = mean spore length (arithmetic average for all spores), W = mean spore width (arithmetic average for all spores), Q = variation in the L/W ratios between the specimens studied, and *n* = a/b (number of spores (a) measured from given number (b) of specimens).

### 2.3. Molecular Phylogeny

The CTAB rapid plant genome extraction kit-DN14 (Aidlab Biotechnologies Co., Ltd., Beijing, P.R. China) was used to obtain genomic DNA from the dried specimens using the manufacturer’s instructions. The nuclear ribosomal ITS region was amplified with primers ITS5 and ITS4 [36]. The nuclear nLSU region was amplified with primer pair LR0R and LR7 (http://lutzonilab.org/nuclear-ribosomal-dna/, accessed on 5 November 2021). The PCR procedure for ITS was as follows: initial denaturation at 95 °C for 3 min, followed by 35 cycles at 94 °C for 40 s, 58 °C for 45 s and 72 °C for 1 min, and a final extension of 72 °C for 10 min. The PCR procedure for nLSU was as follows: initial denaturation at 94 °C for 1 min, followed by 35 cycles at 94 °C for 30 s, 48 °C for 1 min and 72 °C for 1.5 min, and a final extension of 72 °C for 10 min. The PCR products were purified and sequenced at Kunming Tsingke Biological Technology Limited Company (Yunnan Province, P.R. China). All newly generated sequences were deposited in NCBI GenBank (https://www.ncbi.nlm.nih.gov/genbank/, accessed on 20 October 2021) (Table 1).

**Figure 1:**
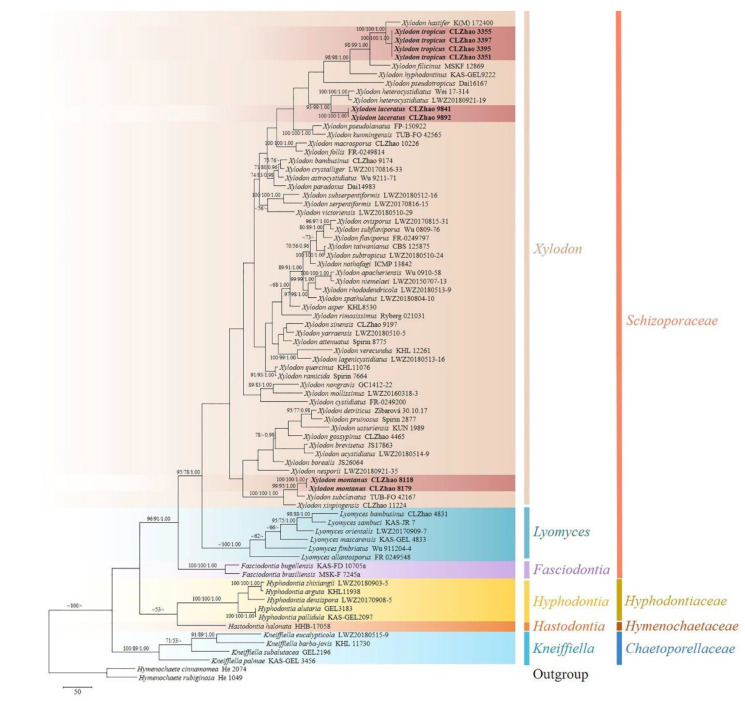
Maximum parsimony strict consensus tree illustrating the phylogeny of *Xylodon* and related genera in Hymenochaetales based on ITS + nLSU sequences.

**Figure 2:**
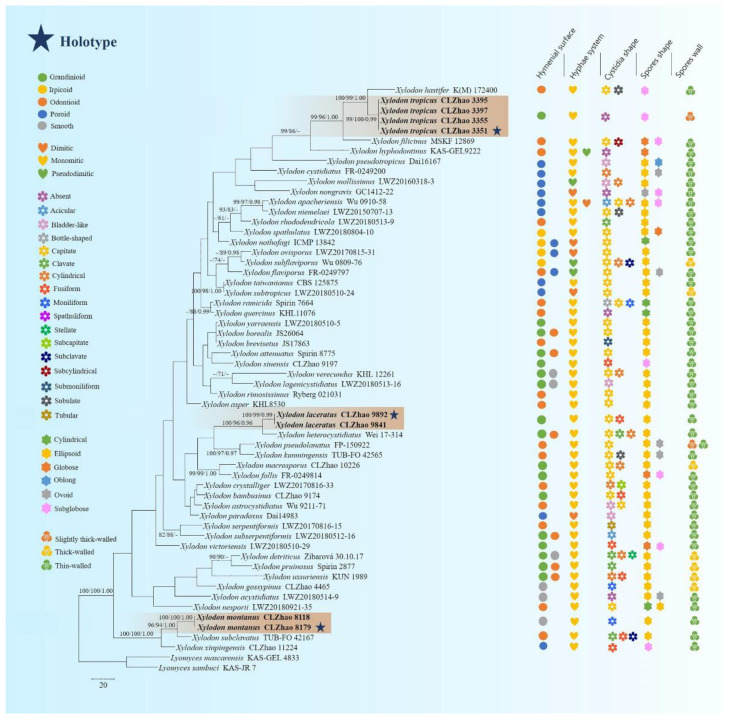
Maximum parsimony strict consensus tree illustrating the phylogeny of three new species in *Xylodon* based on ITS sequences. Branches are labeled with maximum likelihood bootstrap value >70%, parsimony bootstrap value >50%, and Bayesian posterior probabilities >0.95, respectively. The new species are in bold.

The sequences were aligned in MAFFT 7 (https://mafft.cbrc.jp/alignment/server/, accessed on 5 November 2021) using the “G-INS-i” strategy for the ITS and ITS+nLSU dataset. The alignment was adjusted manually using BioEdit [48]. The dataset was aligned first, and then ITS and nLSU sequences were combined with Mesquite version 3.51. Alignment datasets were deposited in TreeBASE (submission ID 29060). ITS + nLSU sequences and ITS-only datasets were used to infer the position of the three new species among *Xylodon* and related taxa. Sequences of *Hymenochaete cinnamomea* (Pers.) Bres. and *H. rubiginosa* (Dicks.) Lév. retrieved from GenBank were used as an outgroup in the ITS+nLSU analysis (Figure 1); sequences of *Lyomyces mascarensis* Riebesehl, Yurchenko & Langer, and *L. sambuca* (Pers.) P. Karst. retrieved from GenBank were used as an outgroup in the ITS-only analysis (Figure 2) [3].

Maximum parsimony (MP), maximum likelihood (ML), and Bayesian inference (BI) analyses were applied to the combined three datasets following a previous study [49], and the tree construction procedure was performed in PAUP* version 4.0b10 [50]. All characters were equally weighted, and gaps were treated as missing data. Using the heuristic search option with TBR branch swapping and 1000 random sequence additions, trees were inferred. Max-trees were set to 5000, branches of zero length were collapsed, and all parsimonious trees were saved. Clade robustness was assessed using bootstrap (BT) analysis with 1000 replicates [51]. Descriptive tree statistics—tree length (TL), consistency index (CI), retention index (RI), rescaled consistency index (RC), and homoplasy index (HI)—were calculated for each maximum parsimonious tree generated. The multiple sequence alignment was also analyzed using maximum likelihood (ML) in RAxML-HPC2 through the Cipres Science Gateway [52]. Branch support (BS) for ML analysis was determined by 1000 bootstrap replicates.

MrModeltest 2.3 [53] was used to determine the best-fit evolution model for each data set for Bayesian inference (BI), which was performed using MrBayes 3.2.7a with a GTR + I + G model of DNA substitution and a gamma distribution rate variation across sites [54]. A total of 4 Markov chains were run for 2 runs from random starting trees for 6 million generations for ITS + nLSU (Figure 1) and 7.5 million generations for ITS (Figure 2) with trees and parameters sampled every 1000 generations. The first one-fourth of all generations were discarded as burn-in. The majority-rule consensus tree of all remaining trees was calculated. Branches were considered as significantly supported if they received maximum likelihood bootstrap value (BS) >70%, maximum parsimony bootstrap value (BT) >70%, or Bayesian posterior probabilities (BPP) >0.95.

## 3. Results

### 3.1. Molecular Phylogeny

The ITS+nLSU dataset (Figure 1) included sequences from 77 fungal specimens representing 71 species. The dataset had an aligned length of 2269 characters. Maximum parsimony analysis yielded 26 equally parsimonious trees (TL = 4541, CI = 0.3510, HI = 0.6490, RI = 0.5827, and RC = 0.2045). The best model for the ITS + nLSU dataset estimated and applied in the Bayesian analysis was GTR + I + G. Bayesian analysis and ML analysis resulted in a similar topology to MP analysis with an average standard deviation of split frequencies = 0.015456 (BI), and the effective sample size (ESS) average ESS (avg ESS) = 791. The phylogram based on the ITS+nLSU rDNA gene regions (Figure 1), including the six genera, *Fasciodontia*, *Hastodontia* (Parmasto) Hjortstam & Ryvarden, *Hyphodontia*, *Kneiffiella*, *Lyomyces*, and *Xylodon*, were divided into four families within Hymenochaetales, which showed that three genera *Fasciodontia*, *Lyomyces,* and *Xylodon* nested into the family Schizoporaceae. Our current three new species clustered into the family Schizoporaceae separated into genus *Xylodon*. *Xylodon laceratus* was a sister to *X. heterocystidiatus* (H.X. Xiong, Y.C. Dai & Sheng H. Wu) Riebesehl, Yurchenko & Langer; *X. montanus* closely related with *X. subclavatus* (H.X. Xiong, Y.C. Dai & Sheng H. Wu) Riebesehl, Yurchenko & Langer and *X. xinpingensis* C.L. Zhao & X. Ma with high supports; *X.* *tropicus* was retrieved as a sister to *X. hastifer* (Hjortstam & Ryvarden) Hjortstam & Ryvarden. 

The ITS-alone dataset (Figure 2) included sequences from 58 fungal specimens representing 53 species. The dataset had an aligned length of 875 characters. Maximum parsimony analysis yielded 5000 equally parsimonious trees (TL = 2054, CI = 0.2965, HI = 0.7035, RI = 0.4580, and RC = 0.1358). The best model for the ITS dataset estimated and applied in the Bayesian analysis was GTR+I+G. Bayesian analysis and ML analysis resulted in a similar topology to the MP analysis with an average standard deviation of split frequencies = 0.028998 (BI), and the effective sample size (ESS) of the average ESS (avg ESS) = 574. 

### 3.2. Taxonomy

**Figure 3:**
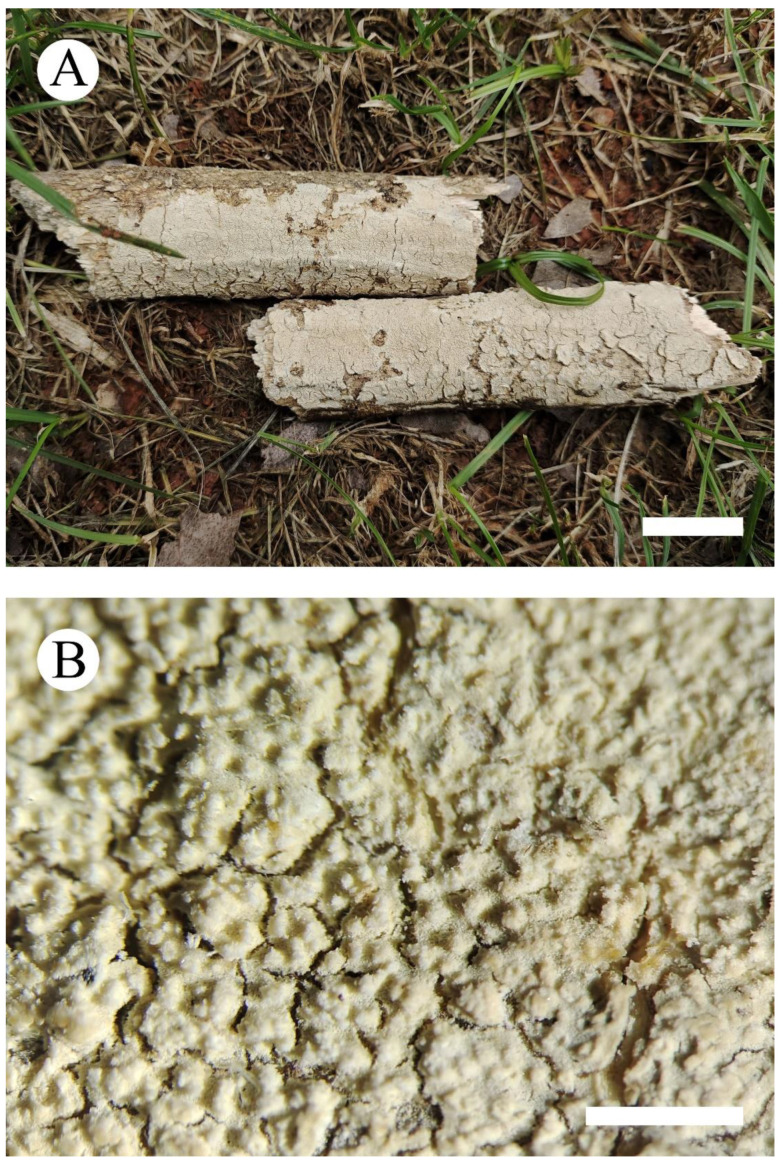
Basidiomata of *Xylodon laceratus* (holotype): the front of the basidiomata (**A**), character hymenophore (**B**). Bars: (**A**) = 2 cm and (**B**) = 1 mm.

**Figure 4:**
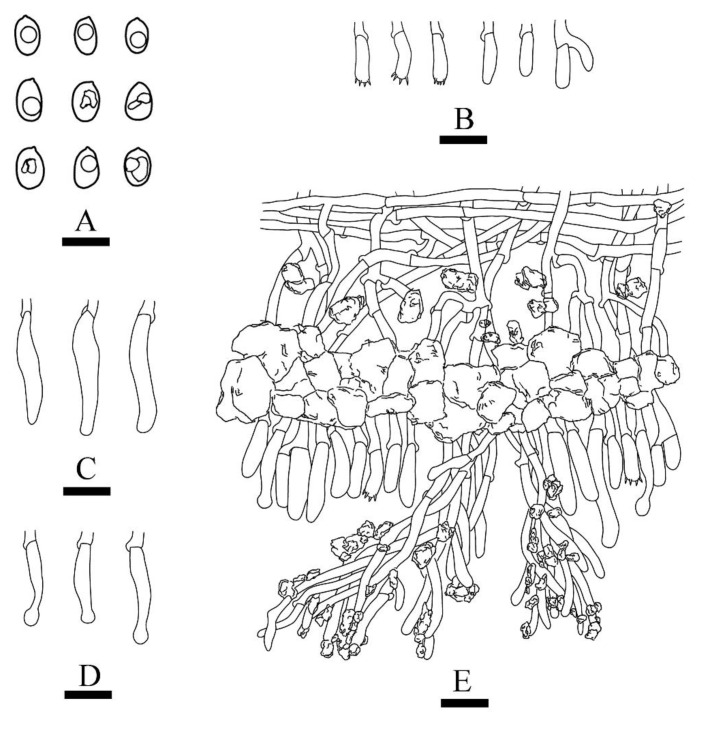
Microscopic structures of *Xylodon laceratus* (holotype): basidiospores (**A**), basidia and basidioles (**B**), fusiform cystidia (**C**), capitate cystidia (**D**), A section of hymenium (**E**). Bars: (**A**) = 5 μm, (**B**)–(**E**) = 10 µm.

***Xylodonlaceratus*** C.L. Zhao, sp. nov. Figure 3 and Figure 4.

MycoBank no.: 842068

**Holotype—**China. Yunnan Province, Puer, Jingdong County, Wuliangshan National Nature Reserve, GPS 22°46′ N, 100°58′ E, altitude 1400 m asl., on the trunk of angiosperm with bark, within the broad-leaved forest, leg. C.L. Zhao, 6 October 2017, CLZhao 9892 (SWFC).

**Etymology—*laceratus*** (Lat.): referring to the cracked hymenophore of the specimens.

**Fruiting body—**Basidiomata annual, resupinate, soft, without odor and taste when fresh, becoming coriaceous when fresh, hard coriaceous upon drying, up to 20 cm long, 3 cm wide, 50–110 µm thick. Hymenial surface grandinioid, aculei up to 0.1 mm long, cream when fresh, cream to buff upon drying, cracking. Sterile margin indistinct, cream, 0.5–1 mm wide, unattached. 

**Hyphal system—**Monomitic, generative hyphae with clamps, colorless, thin-walled, frequently branched, interwoven, 1.8–4.4 µm in diameter, hyphae tight in aculei, IKI–, CB–; tissues unchanged in KOH; subhymenial hyphae densely covered by larger, irregular crystals; a basal layer of hyphae parallel.

**Hymenium—**Cystidia of two types: (1) capitate cystidia rare, smooth, colorless, thin-walled, slightly constricted at the neck, with a globose head, 15.4–24.7 × 3.8–4.7 µm; (2) fusiform cystidia frequently, smooth, colorless, thin-walled, 20.3–26.8 × 5.3–6.4 µm; basidia barreled to clavate, with four sterigmata and a basal clamp connection, 11–17.5 × 3.2–5.5 µm.

**Spores—**Basidiospores ellipsoid, colorless, thin-walled, smooth, with one oil drop inside, IKI–, CB–, (3.7–)3.9–5.3 × 2.6–4.1(–4.8) µm, L = 4.52 µm, W = 3.35 µm, Q = 1.28–1.43 (*n* = 60/2).

**Additional specimen examined—**China, Yunnan Province, Puer, Jingdong County, Wuliangshan National Nature Reserve, GPS 22°46′ N, 100°58′ E, altitude 1400 m asl., on the trunk of angiosperm with bark, within the broad-leaved forest, leg. C.L. Zhao, 6 October 2017, CLZhao 9841 (SWFC).

**Figure 5:**
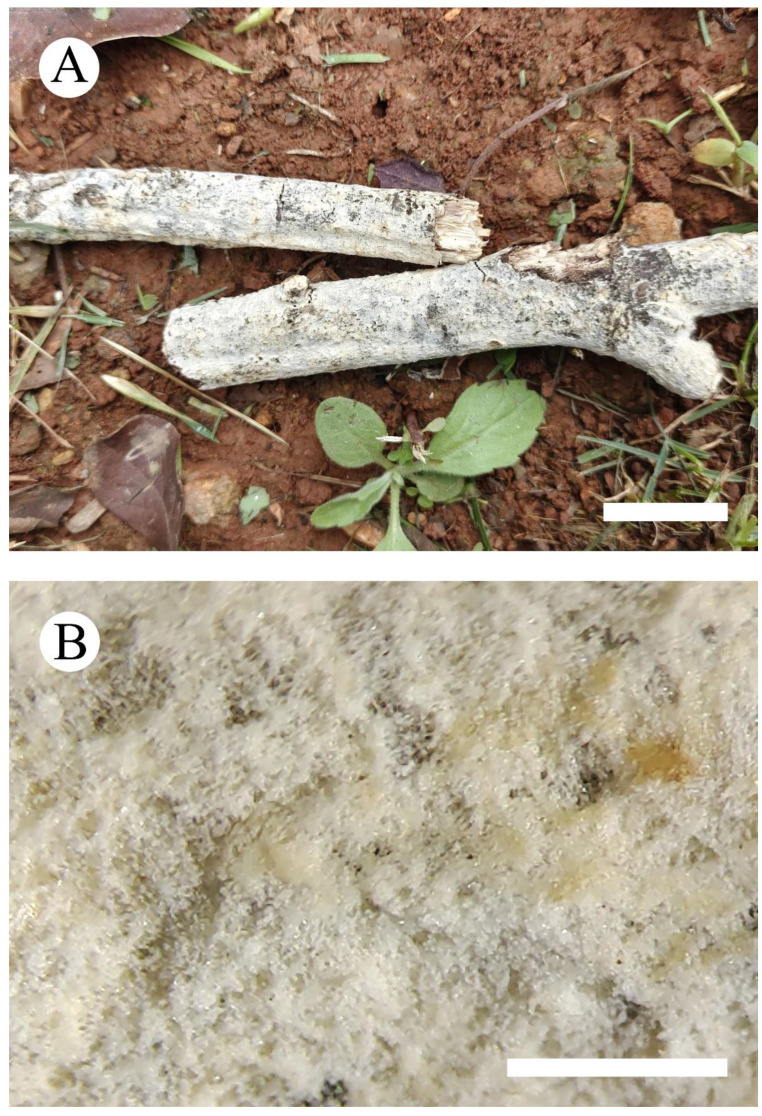
Basidiomata of *Xylodon montanus* (holotype): the front of the basidiomata (**A**), character hymenophore (**B**). Bars: (**A**) = 2 cm and (**B**) = 0.5 mm.

**Figure 6:**
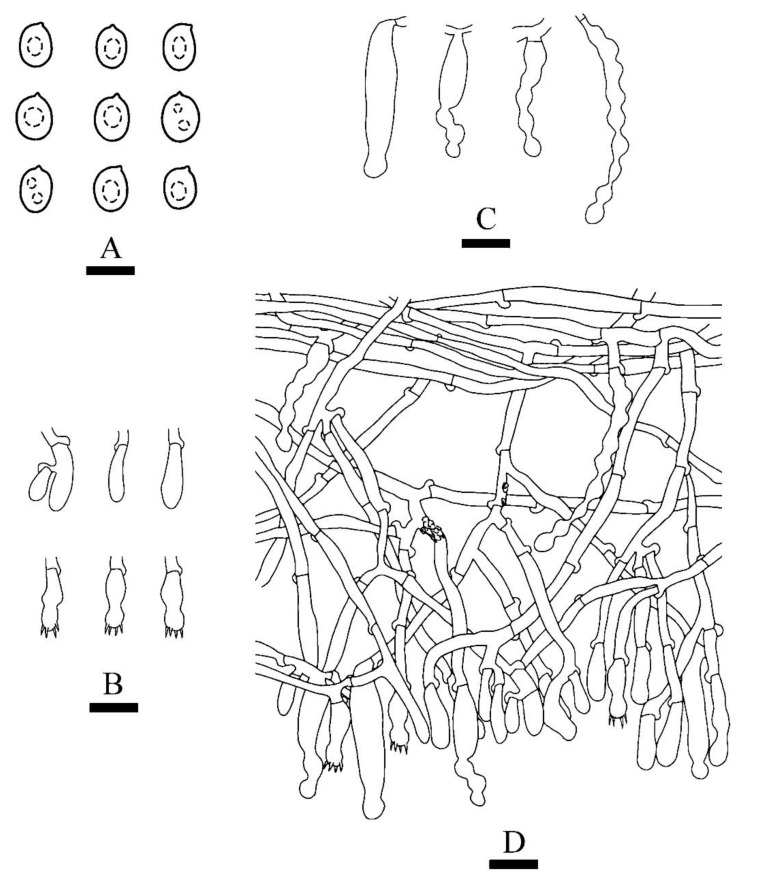
Microscopic structures of *Xylodon montanus* (holotype): basidiospores (**A**), basidia and basidioles (**B**), moniliform cystidia (**C**), A section of hymenium (**D**). Bars: (**A**) = 5 μm, (**B**–**D**) = 10 µm.

***Xylodon montanus*** C.L. Zhao, sp. nov. Figure 5 and Figure 6.

MycoBank no.: 842069

**Holotype—**China. Yunnan Province, Yuxi, Xinping County, Chama Ancient Road Scenic spot, GPS 23°96′ N, 101°51′ E, altitude 2183 m asl., on the angiosperm fallen branch with bark, within the mixed broadleaf-conifer forest, leg. C.L. Zhao, 21 August 2018, CLZhao 8179 (SWFC).

**Etymology—*montanus*** (Lat.): referring for species collected in montane habitat in Yunnan-Guizhou Plateau.

**Fruiting body—**Basidiomata annual, resupinate, woolly when fresh, hard brittle when dry, up to 15 cm long, 3.5 cm wide, 80–130 µm thick. Hymenial surface smooth, white to cream when fresh, cream upon drying. Sterile margin indistinct, white to cream, about 1 mm wide, attached.

**Hyphal system—**Monomitic, generative hyphae with clamps, colorless, thin-walled, frequently branched, interwoven, 1.3–3.7 µm in diameter, IKI–, CB–; tissues unchanged in KOH; a basal layer of hyphae parallel.

**Hymenium—**Cystidia moniliform, numerous, smooth, colorless, thin-walled, slightly constricted at the neck, with a globose head, 19.5–47.6 × 3.6–7.1 µm, rare presence of one globose head; basidia subclavate to clavate with a median constriction, with four sterigmata and a basal clamp connection, 10.1–17.8 × 3.8–5.6 µm.

**Spores—**Basidiospores ellipsoid to broad ellipsoid, colorless, thin-walled, smooth, IKI–, CB–, 3.9–5.3 × 3.2–4.3 µm, L = 4.68 µm, W = 3.61 µm, Q = 1.27–1.32 (*n* = 60/2).

**Additional specimen examined—**China, Yunnan Province, Puer, Zhenyuan County, Ailaoshan, Jinshan Original Forestry, GPS 23°94′ N, 101°52′ E, altitude 2310 m asl., on the angiosperm fallen branch with bark, mixed broadleaf-conifer forest, leg. C.L. Zhao, 21 August 2018, CLZhao 8118 (SWFC).

**Figure 7:**
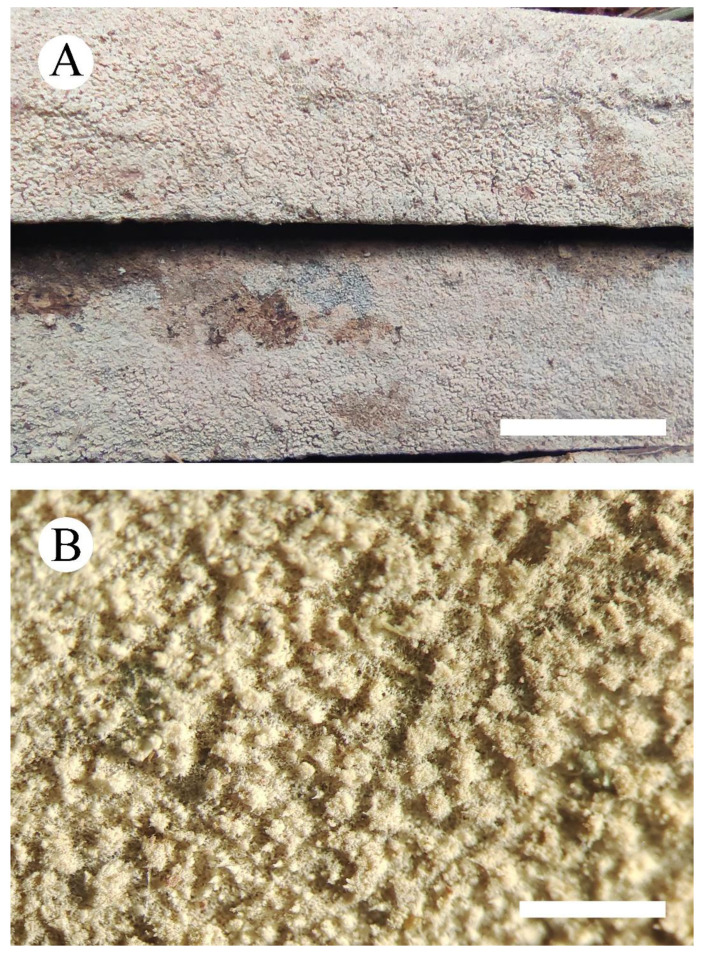
Basidiomata of *Xylodon tropicus* (holotype): the front of the basidiomata (**A**), character hymenophore (**B**). Bars: (**A**) = 1 cm and (**B**) = 1 mm.

**Figure 8:**
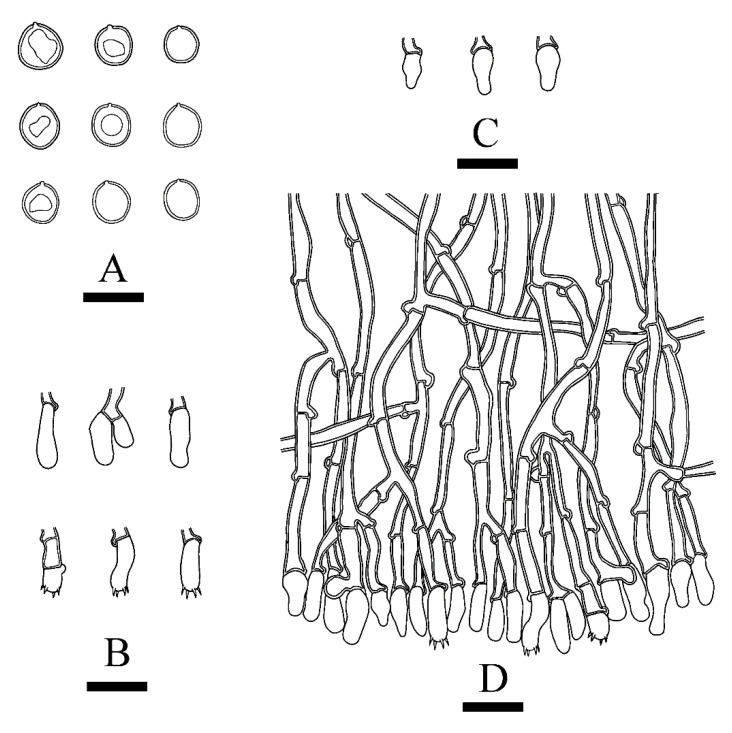
Microscopic structures of *Xylodon tropicus* (holotype): basidiospores (**A**), basidia and basidioles (**B**), and fusiform cystidioles (**C**), A section of hymenium (**D**). Bars: (**A**) = 5 μm, (**B**–**D**) = 10 µm.

***Xylodontropicus*** C.L. Zhao, sp. nov. Figure 7 and Figure 8.

MycoBank no.: 842070

**Holotype—**China. Yunnan Province, Puer, Zhenyuan County, Xieqipo Park, GPS 24°01′ N, 101°10′ E, altitude 1121 m asl., on the branch of dead bamboo, in the bamboo forest, leg. C.L. Zhao, 1 October 2017, CLZhao 3351 (SWFC).

**Etymology—*tropicus*** (Lat.): referring to distribution (tropical zone) of the new species.

**Fruiting body—**Basidiomata annual, resupinate, adnate, coriaceous, without odor and taste when fresh, up to 15 cm long, 6.5 cm wide, 110–250 µm thick. Hymenial surface grandinioid, cream when fresh, buff to pale brown on drying. Sterile margin indistinct, cream to buff, about 1 mm wide, unattached.

**Hyphal system—**Monomitic, generative hyphae with clamps, colorless, thick-walled, 0.4–0.75 µm thick, frequently branched, interwoven, 2.2–3.7 µm in diameter; IKI–, CB–; tissues unchanged in KOH.

**Hymenium—**Cystidia absent and cystidioles fusiform, rare, 7.2–15 × 2.9–4.8 µm; basidia barreled, slightly constricted in the middle to somewhat sinuous, with four sterigmata and a basal clamp, 7.1–13.5 × 2.3–4 µm.

**Spores—**Basidiospores subglobose, colorless, slightly thick-walled, 0.1–0.3 µm thick, smooth, IKI–, CB–, (1.7–)2–4.8 × 1.6–4(–4.4) µm, L = 3.55 µm, W = 3.06 µm, Q = 1.15–1.17 (*n* = 120/4).

**Additional species examined—**China, Yunnan Province, Puer, Zhenyuan County, Xieqipo Park, GPS 24°01′ N, 101°10′ E, altitude 1121 m asl., on the branch of dead bamboo, in the bamboo forest, leg. C.L. Zhao, 1 October 2017, CLZhao 3355, CLZhao 3395, CLZhao 3397 (SWFC).

## 4. Discussion

In their geographical distribution and ecological preferences, the members of *Xylodon* are widespread and primarily wood decomposers, causing a white-rot of woody substrates, which are widely distributed in various forest ecosystems from boreal, temperate, subtropical, to tropical zones [16,55]. Some taxa of *Xylodon* have been collected on rotten trunks and stumps of conifers and angiosperms, bamboo, and ferns [3,12,32,34,44,56,57,58,59,60,61,62,63,64,65,66,67,68,69,70,71,72].

Phylogenetically, the molecular relationships of species belonging to *Hyphodontia* s.l. within Hymenochaetales inferred from the combined dataset of ITS, nLSU, and mt-SSU regions, showed that the phylogeny employed by the dataset strongly supported Hymenochaetales as an independent order, in which seven families, Chaetoporellaceae, Coltriciaceae, Hymenochaetaceae, Neoantrodiellaceae, Nigrofomitaceae, Oxyporaceae, Schizoporaceae were as seven monophyletic lineages, that several genera nested into *Hyphodontia* s.l. were supported as independent genera [3]. In the present study (Figure 1), four related families in the order Hymenochaetales were carried out by the ITS+nLSU analysis, which showed that the six genera, *Fasciodontia*, *Hastodontia*, *Hyphodontia*, *Kneiffiella*, *Lyomyces,* and *Xylodon* nested into related families, and our three new taxa clustered into genus *Xylodon*, belonging to the family Schizoporaceae. 

Molecular phylogeny of the genus *Xylodon* based on the ITS-alone dataset included sequences from 61 fungal specimens representing 55 species, revealed that *X. gossypinus*, *X. macrosporus,* and *X. sinensis* were grouped into three different subclades of *Xylodon*. *X. gossypinus* grouped closely with *X. ussuriensis* Viner; *X. macrosporus* grouped with *X. follis* with high supports, and *X. sinensis* grouped to a clade comprising *X. attenuatus* and *X. yarraensis* with lower supports [32]. In the current study (Figure 1), our three undescribed taxa nested into the genus *Xylodon*, in which *X. laceratus* was a sister to *X. heterocystidiatus*; *X. montanus* closely grouped with *X. subclavatus* and *X. xinpingensis* with a high supports; *X.* *tropicus* was retrieved as a sister to *X. hastifer*. The ITS-based minimum evolution phylogram for *Xylodon* and allied species showed that *X. cystidiatus* (A. David & Rajchenb.) Riebesehl & Langer, *X. hyphodontinus* (Hjortstam & Ryvarden) Riebesehl, Yurchenko & G. Gruhn, *X. serpentiformis* (Langer) Hjortstam & Ryvarden and *X. subclavatus* formed individual lineages in genus *Xylodon*, and two genera *Palifer* and *Odontiopsis* were synonymized under *Xylodon* based on morphological and sequence data [31]; in the same study, *X. erikssonii* (M. Galán & J.E. Wright) Riebesehl & Langer, *X. gamundiae* (Gresl. & Rajchenb.) Riebesehl & Langer, *X. hjortstamii* (Gresl. & Rajchenb.) Riebesehl & Langer*, X. hyphodontinus, X. septocystidiatus* (H.X. Xiong, Y.C. Dai & Sheng H. Wu) Riebesehl & Langer and *X. verecundus* (G. Cunn.) Yurchenko & Riebesehl were proposed as the new combinations [31]. Our new taxa based on the ITS dataset analysis showed that *X*. *tropicus* and *X. hyphodontinus* grouped within the same subclade; both *X. laceratus* and *X. serpentiformis* clustered in the same subclade, but they were distinct; *X. montanus* was closely grouped with *X. subclavatus* with a well supports. However, morphologically, *Xylodon heterocystidiatus* differs from *X. laceratus* by having the membranaceous basidiomata and cylindrical or clavate leptocystidia (34–51 × 6–9 µm) [11]. *X. subclavatus* is different than *X. montanus* by having the cracked basidiomata, odontioid hymenial surface, and subclavate, cylindrical or fusiform cystidia (12–33 × 4.5–8.5 μm) [15]. *X. xinpingensis* is different from *X. montanus* by having the reticulate hymenophore, fusiform cystidia (19.5–31 × 2–6 µm), bigger basidia (18.5–33 × 3–6.5 µm), and subglobose basidiospores (4.5–6 × 3.5–5 µm) [44]. *X. hastifer* could be delimited from *X.* *tropicus* by its whitish, distinctly odontioid hymenophore, and subulate cystidia (40–60 × 6–8 µm) [25].

Morphologically, *Xylodon laceratus* is similar to *X. australis* (Berk.) Hjortstam & Ryvarden, *X*, *gamundiae*, *X. macrosporus*, *X. rhododendricola* Xue W. Wang & L.W. Zhou and *X. subserpentiformis* Xue W. Wang & L.W. Zhou by the cracked hymenophore. However, *Xylodon australis* is diverse from *X. laceratus* by its membranous basidiomata with a brown hymenial surface with an olive tint and subulate, sinuous, or moniliform cystidia (40–60 × 10–20 µm) [22]; *X*. *gamundiae* is different than *X. laceratus* by its suburniform to sinuous basidia (20–30 × 4–5 µm) and cylindrical to subcylindrical basidiospores (6–6.5 × 3–3.5 µm) [56]; *X. macrosporus* differs from *X. laceratus* by its ellipsoid to broad ellipsoid, larger basidiospores (8–10.5 × 7.5–9 µm) [32]; *X. rhododendricola* could be delimited from *X. laceratus* by its odontioid, buff-yellow hymenophore, and usually encrusted with crystals leptocystidia (30–35 × 3–3.5 µm) [3]; *X. subserpentiformis* is distinguishable from *X. laceratus* by its snake-like sinuous tramacystidia (45–50 × 4.5–5.5 µm) and utriform or subclavate basidia (20–25 × 4.5–5.5 µm) [3].

*Xylodon montanus* is similar to *X. anmashanensis* (Yurchenko, H.X. Xiong & Sheng H. Wu) Riebesehl, Yurchenko & Langer, *X. brevisetus* (P. Karst.) Hjortstam & Ryvarden, *X. crassisporus* (Gresl. & Rajchenb.) Hjortstam & Ryvarden, *X. gossypinus* and *X. pumilius* in having the moniliform cystidia. However, *Xylodon anmashanensis* differentiates from *X. montanus* by its cracked, odontioid hymenophore and three kinds of cystidia: hyphoid or subulate cystidia (22–53 × 2.5–4.5 μm), moniliform and submoniliform cystidia (20–60 × 3–5 μm) [15]; *X. brevisetus* is diverse from *X. montanus* by its odontioid hymenophore, clavate to cylindrical basidia (20–25 × 4–5 μm) [25]; *X. crassisporus* could be delimited from *X. montanus* by its odontioid, pale yellow or very pale brown hymenophore, and the capitate cystidia (27–75 × 2–5 μm) [56]; *X. gossypinus* differs from *X. montanus* by its flocculent basidiomata and capitate cystidia (16–23.5 × 2.5–5 µm) [32]; *X. pumilius* is distinguishable from *X. montanus* by its ochraceous hymenophore, capitate cystidia (30–45 × 4–5 μm) and broadly ellipsoid to broadly, thick-walled basidiospores (5–6 × 4–5 μm) [56].

*Xylodontropicus* is similar to *Xylodon damansaraensis* Xue W. Wang & L.W. Zhou, *X.* *septocystidiatus, X. tenellus* Hjortstam & Ryvarden, *X. ussuriensis* Viner and *X*. *yarraensis* in having the grandinioid hymenophore. However, *Xylodon damansaraensis* distinguishes from *X.* *tropicus* by its white hymenophore, clavate-sinuous to submoniliform cystidia (35–40 × 6–7 µm) and narrowly ellipsoid basidiospores (5.2–5.7 × 2.3–3.1 µm) [3]; *X. septocystidiatus* differentiates from *X.* *tropicus* by its pale buff hymenophore and subcylindrical basidia with subuniform constriction (16–22 × 3 4.5–5 µm) [12]; *X. tenellus* is diverse from *X.* *tropicus* by its whitish hymenophore, capitate cystidia (30–60 µm long) and thin-walled basidiospores (4 × 4.5 µm) [28]; *X. ussuriensis* could be delimited from *X.* *tropicus* by its pale ochraceous hymenophore, three types of cystidia: capitate cystidia (71.0–188.9 × 5.7–9.4 μm), astrocystidia (15–17 × 4.5–4.8 μm), fusoid to cylindrical or submoniliform cystidia (40.0–84.0 × 5.0–9.0 μm), and ellipsoid to broadly ellipsoid basidiospores (5.1–6.0 × 3.8–4.6 μm) [27]; *X. yarraensis* is different from *X.* *tropicus* by its capitate cystidia (25–30 × 2.5–3.5 µm) and ellipsoid basidiospores (4.5–5.3) × 3.1–3.8 µm) [3].

Miettinen et al. [71] analyzed a higher-level phylogenetic classification of polypores and showed that the macromorphology of fruiting bodies and hymenophore construction did not reflect monophyletic groups. The current phylogeny shows that the morphological characters do not follow the phylogenetic group on different taxa in this genus. However, several characters lead the key role, e.g., the hyphal system monomitic, basidiospores ellipsoid, and thin-walled (Figure 2).

So far, 33 taxa of *Xylodon* were recorded in China [3,22,24,27,31,32,37,44,72]. Based on the morphological and phylogenetic study of *Xylodon* presented here, all of these can be delimited from our three new species (Figure 1 and Figure 2).

## Figures and Tables

**Table 1:** List of species, specimens, and GenBank accession numbers of sequences used in this study.

Species Name	Specimen No.	GenBank Accession No.		References	Country
		ITS	nLSU		
*Fasciodontia brasiliensis*	MSK-F 7245a	MK575201	MK598734	[37]	Brazil
*F. bugellensis*	KAS-FD 10705a	MK575203	MK598735	[37]	France
*Hastodontia halonata*	HHB-17058	MK575207	MK598738	[37]	Mexico
*Hymenochaete cinnamomea*	He 2074	KU975460	KU975500	Unpublished	China
*Hym. rubiginosa*	He 1049	JQ716407	JQ279667	[38]	China
*Hyphodontia alutaria*	GEL3183	DQ340318	DQ340373	Unpublished	Germany
*Hyp. arguta*	KHL11938	EU118632	EU118633	[39]	Sweden
*Hyp. pallidula*	KAS-GEL2097	DQ340317	DQ340372	Unpublished	Germany
*Hyp. densispora*	LWZ20170908-5	MT319426	MT319160	[3]	China
*Hyp. zhixiangii*	LWZ20180903-5	MT319423	MT319158	[3]	China
*Kneiffiella barba-jovis*	KHL 11730	DQ873609	DQ873610	[31]	Sweden
*K. eucalypticola*	LWZ20180515-9	MT319411	MT319143	[3]	Australia
*K. palmae*	KAS-GEL 3456	DQ340333	DQ340369	[37]	China
*K. subalutacea*	GEL2196	DQ340341	DQ340362	[37]	Norway
*Lyomyces allantosporus*	FR 0249548	KY800397	KY795963	[40]	Réunion
*L. bambusinus*	CLZhao 4831	MN945968	MW264919	[41]	China
*L. fimbriatus*	Wu 911204-4	MK575210	MK598740	[37]	China
*L. mascarensis*	KAS-GEL 4833	KY800399	KY795964	[37]	Réunion
*L. orientalis*	LWZ20170909-7	MT319436	MT319170	[3]	China
*L. sambuci*	KAS-JR 7	KY800402	KY795966	[40]	Germany
*Xylodon acystidiatus*	LWZ20180514-9	MT319474	MT319211	[3]	Australia
*X. apacheriensis*	Wu 0910-58	KX857797	KX857822	[42]	China
*X. asper*	KHL8530	AY463427	AY586675	[43]	Sweden
*X. astrocystidiatus*	Wu 9211-71	JN129972	JN129973	[16]	China
*X. attenuatus*	Spirin 8775	MH324476		[27]	USA
*X. australis*	LWZ20180509-8	MT319503		[3]	China
*X. bambusinus*	CLZhao 9174	MW394657	MW394650	[44]	China
*X. borealis*	JS26064	AY463429	AY586677	[43]	Norway
*X. brevisetus*	JS17863	AY463428	AY586676	[43]	Norway
*X. crystalliger*	LWZ20170816-33	MT319521	MT319269	[3]	China
*X. cystidiatus*	FR-0249200	MH880195	MH884896	[31]	Réunion
*X. damansaraensis*	LWZ20180417-23	MT319499		[3]	Malaysia
*X. detriticus*	Zíbarová 30.10.17	MH320793	MH651372	[27]	Czech Republic
*X. filicinus*	MSK F 12869	MH880199	NG067836	[31]	China
*X. flaviporus*	FR-0249797	MH880201	MH884901	[31]	Réunion
*X. follis*	FR-0249814	MH880204	MH884902	[31]	Réunion
*X. gossypinus*	CLZhao 4465	MZ663803	MZ663812	[32]	China
*X. hastifer*	K(M) 172400	NR166558		[22]	USA
*X. heterocystidiatus*	LWZ20180921-19	MT319676	MT319266	[3]	Australia
*X. heterocystidiatus*	Wei 17-314	MT731753	MT731754	Unpublished	China
*X. hyphodontinus*	KAS-GEL9222	MH880205	MH884903	[31]	Kenya
*X. kunmingensis*	TUB-FO 42565	MH880198	MH884898	[31]	China
*X. laceratus*	CLZhao 9841	OL619257	OL619265	Present study	China
*X. laceratus*	CLZhao 9892 [T]	OL619258	OL619266	Present study	China
*X. lagenicystidiatus*	LWZ20180513-16	MT319634	MT319368	[3]	Australia
*X. lenis*	Wu890714-3	KY081802		[22]	China
*X. macrosporus*	CLZhao 10226	MZ663809	MZ663817	[32]	China
*X. mollissimus*	LWZ20160318-3	KY007517	MT319347	[3]	China
*X. montanus*	CLZhao 8118	OL619259	OL619267	Present study	China
*X. montanus*	CLZhao 8179 [T]	OL619260	OL619268	Present study	China
*X. nesporii*	LWZ20180921-35	MT319655	MT319238	[3]	China
*X. niemelaei*	LWZ20150707-13	MT319630	MT319365	[3]	China
*X. nongravis*	GC1412-22	KX857801	KX857818	[42]	China
*X. nothofagi*	ICMP 13842	AF145583		[45]	China
*X. ovisporus*	LWZ20170815-31	MT319666	MT319346	[3]	China
*X. papillosus*	CBS 114.71	MH860026		[46]	Netherlands
*X. paradoxus*	Dai14983	MT319519	MT319267	[3]	China
*X. pruinosus*	Spirin 2877	MH332700		[27]	Estonia
*X. pseudolanatus*	FP-150922	MH880220	NG067837	[31]	Belize
*X. pseudotropicus*	Dai16167	MT319509	MT319255	[3]	China
*X. quercinus*	KHL11076	KT361633	AY586678	[43]	Sweden
*X. ramicida*	Spirin 7664	NR138013		Unpublished	USA
*X. rhododendricola*	LWZ20180513-9	MT319621	MT319357	[3]	Australia
*X. rimosissimus*	Ryberg 021031	DQ873627	DQ873628	[47]	Sweden
*X. serpentiformis*	LWZ20170816-15	MT319673	MT319218	[3]	China
*X. sinensis*	CLZhao 9197	MZ663810	MZ663818	[32]	China
*X. spathulatus*	LWZ20180804-10	MT319646	MT319354	[3]	China
*X. subclavatus*	TUB-FO 42167	MH880232		[31]	China
*X. subflaviporus*	Wu 0809-76	KX857803	KX857815	[42]	China
*X. subserpentiformis*	LWZ20180512-16	MT319486	MT319226	[3]	Australia
*X. subtropicus*	LWZ20180510-24	MT319541	MT319308	[3]	China
*X. taiwanianus*	CBS 125875	MH864080	MH875537	[46]	Netherlands
*X. tropicus*	CLZhao 3351 [T]	OL619261	OL619269	Present study	China
*X. tropicus*	CLZhao 3355	OL619262		Present study	China
*X. tropicus*	CLZhao 3395	OL619263	OL619270	Present study	China
*X. tropicus*	CLZhao 3397	OL619264	OL619271	Present study	China
*X. ussuriensis*	KUN 1989	NR166241		Unpublished	USA
*X. verecundus*	KHL 12261	DQ873642	DQ873643	[47]	Sweden
*X. victoriensis*	LWZ20180510-29	MT319487	MT319228	[3]	Australia
*X. xinpingensis*	CLZhao 11224	MW394662	MW394654	[44]	China
*X. yarraensis*	LWZ20180510-5	MT319639	MT319378	[3]	Australia
*X. yunnanensis*	LWZ20180922-47	MT319660		[3]	China

[T] is shown type material, holotype.

## Data Availability

Publicly available datasets were analyzed in this study. This data can be found here: [https://www.ncbi.nlm.nih.gov/; https://www.mycobank.org/page/Simple%20names%20search; http://purl.org/phylo/treebase, submission ID 29060; accessed on 30 November 2021].

## References

[1] Hibbett D.S., Bauer R., Binder M., Giachini A.J., Hosaka K., Justo A., Larsson E., Larsson K.H., Lawrey J.D., Miettinen O. (2014). 14: Agaricomycetes. Systematics and Evolution.

[2] Dai Y.C. (2011). A revised checklist of corticioid and hydnoid fungi in China for 2010. Mycoscience.

[3] Wang X.W., May T.W., Liu S.L., Zhou L.W. (2021). Towards a Natural Classification of *Hyphodontia* Sensu Lato and the Trait Evolution of Basidiocarps within Hymenochaetales (Basidiomycota). J. Fungi.

[4] Jülich W. (1981). Higher taxa of Basidiomycetes. Bibl. Mycol..

[5] Gray S.F. (1821). A Natural Arrangement of British Plants.

[6] Bernicchia A., Gorjón S.P. (2010). Fungi Europaei 12: Corticiaceae s.l..

[7] Wu S.H. (1990). The Corticiaceae (Basidiomycetes) subfamilies Phlebioideae, Phanerochaetoideae and Hyphodermoideae in Taiwan. Ann. Bot. Fenn..

[8] Wu S.H. (2000). Studies on *Schizopora flavipora* s.l., with special emphasis on specimens from Taiwan. Mycotaxon.

[9] Wu S.H. (2001). Three new species of *Hyphodontia* with poroid hymenial surface. Mycologia.

[10] Wu S.H. (2006). *Hyphodontia tubuliformis*, a new species from Taiwan. Mycotaxon.

[11] Xiong H.X., Dai Y.C., Wu S.H. (2009). Three new species of *Hyphodontia* from Taiwan. Mycol. Prog..

[12] Xiong H.X., Dai Y.C., Wu S.H. (2010). Two new species of *Hyphodontia* from China. Mycologia.

[13] Dai Y.C. (2012). Polypore diversity in China with an annotated checklist of Chinese polypores. Mycoscience.

[14] Lee I.S., Langer E. (2012). New records of *Hyphodontia* species from Taiwan. Nova Hedwig..

[15] Yurchenko E., Xiong H.X., Wu S.H. (2013). Four new species of *Hyphodontia* (*Xylodon* s.s. Hjortstam & Ryvarden, Basidiomycota) from Taiwan. Nowa Hedwig..

[16] Yurchenko E., Wu S.H. (2014). *Hyphoderma formosanum* sp. nov. (Meruliaceae, Basidiomycota) from Taiwan. Sydowia.

[17] Zhao C.L., Cui B.K., Dai Y.C. (2014). Morphological and molecular identification of two new species of *Hyphodontia* (Schizoporaceae, Hymenochaetales) from southern China. Cryptogam. Mycol..

[18] Chen J.J., Zhou L.W., Ji X.H., Zhao C.L. (2016). *Hyphodontia dimitica* and *H. subefibulata* spp. nov. (Schizoporaceae, Hymenochaetales) from southern China based on morphological and molecular characters. Phytotaxa.

[19] Chen C.C., Wu S.H., Chen C.Y. (2018). *Xylodon subflaviporus* sp. nov. (Hymenochaetales, Basidiomycota) from East Asia. Mycoscience.

[20] Kan Y.H., Gafforov Y., Li T., Zhou L.W. (2017). *Hyphodontia zhixiangii* sp. nov. (Schizoporaceae, Basidiomycota) from Uzbekistan. Phytotaxa.

[21] Kan Y.H., Qin W.M., Zhou L.W. (2017). *Hyphodontia mollissima* sp. nov. (Schizoporaceae, Hymenochaetales) from Hainan, southern China. Mycoscience.

[22] Riebesehl J., Langer E. (2017). *Hyphodontia* s.l. (Hymenochaetales, Basidiomycota): 35 new combinations and new keys to all 120 current species. Mycol. Prog..

[23] Wang M., Chen Y.Y. (2017). Phylogeny and taxonomy of the genus *Hyphodontia* (Hymenochaetales, Basidiomycota) in China. Phytotaxa.

[24] Shi Z.W., Wang X.W., Zhou L.W., Zhao C.L. (2019). *Xylodon kunmingensis* sp. nov. (Hymenochaetales, Basidiomycota) from southern China. Mycoscience.

[25] Hjortstam K., Ryvarden L. (2009). A checklist of names in *Hyphodontia* sensu stricto-sensu lato and *Schizopora* with new combinations in *Lagarobasidium*, *Lyomyces*, *Kneiffiella*, *Schizopora*, and *Xylodon*. Syn. Fungorum.

[26] Kuntze O. (1898). Iridaceae. Revisio Generum Plantarum.

[27] Viner I., Spirin V., Zíbarová L., Larsson K.H. (2018). Additions to the taxonomy of *Lagarobasidium* and *Xylodon* (Bymenochaetales, Basidiomycota). Mycokeys.

[28] Hjortstam K., Ryvarden L. (2007). Studies in corticioid fungi from Venezuela III (Basidiomycotina, Aphyllophorales). Syn. Fungorum.

[29] Chevallier F.F. (1826). Flore Générale des Environs de Paris.

[30] Tura D.A., Zmitrovich I.V., Wasser S.P., Spirin W.A., Nevo E. (2011). Biodiversity of the Heterobasidiomycetes and Non-Gilled Hymenomycetes (Former Aphyllophorales) of Israel.

[31] Riebesehl J., Yurchenko E., Nakasone K.K., Langer E. (2019). Phylogenetic and morphological studies in *Xylodon* (Hymenochaetales, Basidiomycota) with the addition of four new species. MycoKeys.

[32] Luo K.Y., Qu M.H., Zhao C.L. (2021). Additions to the knowledge of corticioid *Xylodon* (Schizoporaceae, Hymenochaetales): In-troducing three new *Xylodon* species from southern China. Diversity.

[33] James T.Y., Stajich J.E., Hittinger C.T., Rokas A. (2020). Toward a fully resolved fungal tree of life. Annu. Rev. Microbiol..

[34] Yurchenko E., Wu S.H. (2013). Three new species of *Hyphodontia* with peg-like hyphal aggregations. Mycol. Prog..

[35] Petersen J.H. (1996). Farvekort. The Danish Mycological Society’s Colour-Chart.

[36] White T.J., Bruns T., Lee S., Taylor J. (1990). Amplification and direct sequencing of fungal ribosomal RNA genes for phylogenetics. PCR Protoc. A Guide Methods Appl..

[37] Yurchenko E., Riebesehl J., Langer E.J. (2020). *Fasciodontia* gen. nov. (Hymenochaetales, Basidiomycota) and the taxonomic status of Deviodontia. Mycol. Prog..

[38] He S.H., Li H.J. (2013). *Pseudochaete latesetosa* and *P. subrigidula* spp. nov. (Hymenochaetales, Basidiomycota) from China based on morphological and molecular characters. Mycol. Prog..

[39] Larsson K.H. (2007). Re-thinking the classification of corticioid fungi. Mycol. Res..

[40] Yurchenko E., Riebesehl J., Langer E. (2017). Clarification of *Lyomyces sambuci* complex with the descriptions of four new species. Mycol. Prog..

[41] Chen J.Z., Zhao C.L. (2020). Morphological and molecular identification of four new resupinate species of *Lyomyces* (Hymenochaetales) from southern China. MycoKeys.

[42] Chen C.C., Wu S.H., Chen C.Y. (2017). Three new species of *Hyphodontia* s.l. (Basidiomycota) with poroid or raduloid hymenophore. Mycol. Prog..

[43] Larsson K.H., Larsson E., Kõljalg U. (2004). High phylogenetic diversity among corticioid homobasidiomycetes. Mycol. Res..

[44] Ma X., Zhao C.L. (2021). *Xylodon bambusinus* and *X. xinpingensis* spp. nov. (Hymenochaetales) from southern China. Phytotaxa.

[45] Paulus B., Hallenberg N., Buchanan P.K., Chambers G.K. (2000). A phylogenetic study of the genus *Schizopora* (Basidiomycota) based on ITS DNA sequences. Mycol. Res..

[46] Vu D., Groenewald M., de Vries M., Gehrmann T., Stielow B., Eberhardt U., Al-Hatmi A., Groenewald J.Z., Cardinali G., Houbraken J. (2019). Large-scale generation and analysis of filamentous fungal DNA barcodes boosts coverage for kingdom fungi and reveals thresholds for fungal species and higher taxon delimitation. Stud. Mycol..

[47] Larsson K.H., Parmasto E., Fischer M., Langer E., Nakasone K.K., Redhead S.A. (2006). Hymenochaetales: A molecular phylogeny for the hymenochaetoid clade. Mycologia.

[48] Hall T.A. (1999). Bioedit: A user-friendly biological sequence alignment editor and analysis program for windows 95/98/NT. Nucleic Acids Symp. Ser..

[49] Zhao C.L., Wu Z.Q. (2017). *Ceriporiopsis kunmingensis* sp. nov. (Polyporales, Basidiomycota) evidenced by morphological characters and phylogenetic analysis. Mycol. Prog..

[50] Swofford D.L. (2002). PAUP: Phylogenetic Analysis Using Parsimony (and Other Methods).

[51] Felsenstein J. (1985). Confidence intervals on phylogenetics: An approach using bootstrap. Evolution.

[52] Miller M.A., Pfeiffer W., Schwartz T. (2012). The CIPRES Science Gateway: Enabling high-impact science for phylogenetics researchers with limited resources. Assoc. Comput. Mach..

[53] Nylander J.A.A. (2004). MrModeltest v2. Program Distributed by the Author.

[54] Ronquist F., Teslenko M., van der Mark P., Ayres D.L., Darling A., Hohna S., Larget B., Liu L., Suchard M.A., Huelsenbeck J.P. (2012). Mrbayes 3.2: Efficient bayesian phylogenetic inference and model choice across a large model space. Syst. Biol..

[55] Eriksson J., Ryvarden L. (1976). The Corticiaceae of North Europe. Syn. Fungorum.

[56] Greslebin A.G., Rajchenberg M. (2000). The genus *Hyphodontia* in the Patagonian Andes forest of Argentina. Mycologia.

[57] Kotiranta H., Saarenoksa R. (2000). Three new species of *Hyphodontia* (Coritciaceae). Ann. Bot. Fenn..

[58] Boidin J., Gilles G. (2003). Homobasidiomycètes Aphyllophorales non porés à basides dominantes à 2 (3) stérigmates. Bull. Trimest. Soc. Mycol. Fr..

[59] Burdsall H.H., Nakasone K.K., Freeman G.W. (1981). New species of *Gloeocystidiellum* (Corticiaceae) from the southeastern United-States. Syst. Bot..

[60] Langer E. (1994). Die Gattung *Hyphodontia* John Eriksson. Bibl. Mycol..

[61] Nordén B., Appelquist T., Lindahl B., Henningsson M. (1999). Cubic rot fungi–corticioid fungi in highly brown rotted spruce stumps. Mycol. Helv..

[62] Hjortstam K., Ryvarden L., Itturiaga T. (2005). Studies in corticioid fungi from Venezuela II (Basidiomycotina, Aphyllophorales). Syn. Fungorum.

[63] Jo J.W., Kwag Y.N., Kim N.K., Oh S.O., Kim C.S. (2018). A-33: Newly recorded macrofungal species (*Xylodon flaviporus*) in Dokdo, Republic of Korea. KSM Newsl..

[64] Gilbertson R.L., Ryvarden L. (1987). North American Polypores 1–2.

[65] Núñez M., Ryvarden L. (2001). East Asian polypores 2. Syn. Fungorum.

[66] Ryvarden L., Melo I. (2014). Poroid fungi of Europe. Syn. Fungorum.

[67] Yurkov A., Wehde T., Kahl T., Begerow D. (2012). Aboveground deadwood deposition supports development of soil yeasts. Diversity.

[68] Girometta C.E., Bernicchia A., Baiguera R.M., Bracco F., Buratti S., Cartabia M., Picco A.M., Savino E. (2020). An italian research culture collection of wood decay fungi. Diversity.

[69] Van Bael S.A. (2020). Fungal diversity. Diversity.

[70] Ogura-Tsujita Y., Tetsuka K., Tagane S., Kubota M., Anan S., Yamashita Y., Tone K., Yukawa T. (2021). Differing life-history strategies of two mycoheterotrophic orchid species associated with leaf litter- and wood-decaying fungi. Diversity.

[71] Miettinen O., Spirin V., Vlasák J., Rivoire B., Stenroos S., Hibbett D. (2016). Polypores and genus concepts in Phanerochaetaceae (Polyporales, Basidiomycota). MycoKeys.

[72] Gafforov Y., Riebesehl J., Ordynets A., Langer E., Yarasheva M., Ghobad-Nejhad M., Zhou L.W., Wang X.W., Gugliotta A.D.M. (2017). *Hyphodontia* (Hymenochaetales, Basidiomycota) and similar taxa from Central Asia. Botany.

